# Protective Effects of Angong Niuhuang Pill on Early Atherosclerosis in ApoE^−/−^ Mice by Reducing the Inflammatory Response

**DOI:** 10.1155/2019/9747212

**Published:** 2019-05-20

**Authors:** Yushuang Chai, Zhen Yin, Qinghong Fan, Zhe Zhang, Kaihe Ye, Yimin Xu, Wei Xiao, Xiaomeng Chai, Tao Zhu, Hong Nie

**Affiliations:** ^1^Guangzhou Baiyunshan Zhongyi Pharmaceutical Co., Ltd, Guangzhou 510530, China; ^2^Guangdong Province Key Laboratory of Pharmacodynamic Constituents of TCM and New Drugs Research, College of Pharmacy, Jinan University, 601 Huangpu Avenue West, Guangzhou, Guangdong, China; ^3^International Cooperative Laboratory of Traditional Chinese Medicine Modernization and Innovative Drug Development of Chinese Ministry of Education (MOE), College of Pharmacy, Jinan University, 601 Huangpu Avenue West, Guangzhou, China

## Abstract

Atherosclerosis (AS) is the primary cause of cardiocerebrovascular disease, and inflammation is responsible for the initiation of its pathogenesis. Therefore, targeting inflammatory pathways to prevent AS progression is an ideal strategy. Angong Niuhuang pill (ANP) is a well-known traditional Chinese medicine and has been widely used for thousands of years to treat central nervous system and cardiovascular diseases. In this study, we investigated the role of ANP in reducing inflammation during early AS, using a high-fat diet-induced ApoE^−/−^ mouse model of AS. Compared to those with simvastatin, ANP had no significant effect on serum triglyceride, low-density lipoprotein, and high-density lipoprotein levels. However, it effectively inhibited splenic and vascular inflammation. This agent also reduced the Th17/CD4^+^T ratio and mRNA expression of* IL-6* and increased the Treg/CD4^+^T ratio and mRNA expression of* TGF-β1*. Thus, ANP restored Th17/Treg homeostasis in the spleen. It also regulated pro- and anti-inflammatory cytokine expression in the aorta in a similar manner. Further, it downregulated the expression of chemokine receptors (CCR2, CXCR3), their ligands (MCP-1, MCP-2, and MCP-3), and cell adhesion molecules (VCAM-1, ICAM-1) in arterial vessels. These results indicate that ANP can ameliorate the development of early AS, mainly by reducing inflammation instead of acting as an antihyperlipidemic drug.

## 1. Introduction

Cardiovascular disease (CVD), a global chronic disease, is responsible for approximately one-third of all deaths worldwide [1, 2]. According to a report released by the World Economic Forum and the Harvard School of Public Health in 2011, the cost of CVD treatment comprised approximately 863 billion dollars of the global economy in 2010, and this number is expected to rise to 1044 billion dollars by 2030. According to an estimation by the World Bank and an evaluation of purchasing power parity in China, the economic benefits from decreasing the CVD-associated death rate by 1% from 2010 to 2040 will be equivalent to 68% of the GDP of the country in 2010 (i.e., more than 10.7 trillion dollars) [3]. Therefore, innovative ways to prevent or treat CVD are vital, as this disorder affects the wellbeing and social stability of individuals.

Most aspects of CVD and its complications are driven by atherosclerosis (AS) [4]. Over the last three decades, AS has been widely accepted as a chronic inflammatory disease. Increased T lymphocyte subset counts and arterial wall inflammation function as the main underlying pathogenic factors of this disease [5–7]. In the early stage of lesion formation, CD4^+^ T cells are the most abundant T cell subset in the atherosclerotic plaques. When stimulated by different cytokines and various environment conditions, CD4^+^ T cells can be differentiated into T helper cell 1 (Th1), Th2, T regulatory (Treg), and Th17 cells [8]. Frostegard et al. demonstrated the upregulation of Th1 and proinflammatory cytokines (interleukin (IL)-2 and -7) in local atherosclerotic plaques and circulating lymphocytes, whereas anti-inflammatory cytokines were downregulated [9]. These results suggest that Th1/Th2 imbalances are important for lesion formation and early AS development [10–12]. In recent years, Th17/Treg cell ratios have been recognized as a different balance from Th1/Th2 proportions. Treg cells play an anti-inflammatory role by secreting anti-inflammatory cytokines such as IL-10 and TGF-*β* [13]. Th17, in contrast, can promote inflammation by releasing the proinflammatory cytokines IL-17 and IL-6 [14]. Therefore, the Th17/Treg balance is a prominent factor for the prevention or development of immune-related diseases. Clinical studies have found the Th17/Treg balance in patients corresponds to acute coronary syndrome [15]. Further, Jiangjiao Xie et al. confirmed an imbalance in Th17/Treg cells during AS in an ApoE^−/−^ mouse model, which implied that such events are crucial for the formation and progression of AS [5].

Likewise, leukocyte accumulation also plays important roles in the growth of atherosclerotic lesions. Monocytes and neutrophils can be recruited through interactions with activated endothelial cells. During this interaction, chemokines and their respective receptors, endothelial adhesion molecules, are upregulated. Chemokines, a class of small heparin-binding proteins behave as a chemoattractant for leukocytes [16]. Further, they regulate the trafficking, migration, growth, and activation of leukocytes to inflammatory and injured tissues by binding their cognate receptors, seven-transmembrane G protein-coupled-receptors [17]. Presently, approximately 50 chemokine ligands and 20 chemokine receptors have been identified. Based on differences in cysteine arrangements at the N-terminus, chemokines can be classified into four main subfamilies including C, CC, CXC, and CX3C (C-Cysteine, X-any amino acid). Their corresponding receptors can also be classified as CR, CCR, CXCR, and CX3CR, respectively [18, 19]. Many cross interactions can also occur between chemokines and chemokine receptors. Thus, one chemokine can be recognized by more than one chemokine receptor and vice versa [17]. Previous studies indicate that three closely related and well-known chemokines, namely, monocyte chemoattractant protein (MCP) 1, 2, and 3 (belonging to the CC subfamily), as well as their cognate receptors, CCR2 and CXCR3, play pivotal roles in the pathogenesis of early AS. Gu et al. knocked out MCP-1 in low-density lipoprotein (LDL) receptor-deficient mice, and this resulted in an 83% decrease in lipid deposition throughout the aortas. This decrease indicated that MCP-1 plays a crucial role in AS [20]. Moreover, Aiello et al. found that the overexpression of MCP-1 could accelerate AS progression by increasing the number of macrophages and the accumulation of oxidized lipids [21]. In addition, Dawson showed that CCR2 knockout in ApoE^−/−^ mice results in a mean 3-fold reduction in the lesion area of the aorta as compared to that in control animals [22]. The deletion of individual chemokines or chemokine receptors results in only the partial inhibition of AS development. However, the combined inhibition of MCP-1, CX3CR1, and CCR5 almost completely abolishes AS [23]. This difference implies that inhibiting multiple inflammatory targets is crucial for the treatment of this disease. Cell adhesion molecule (CAM) is a type of transmembrane receptor that binds other cells and the extracellular matrix. Intracellular adhesion molecules (ICAMs) and vascular adhesion molecules (VCAMs) are two types of well-studied CAMs and share identical structures and functions [24]. They participate in the regulation of atherogenesis by promoting interactions with leukocyte integrins [25]. Nakashima et al. analyzed the expression levels of ICAM-1 and VCAM-1 in ApoE^−/−^ mice and observed the upregulation of both molecules, indicating their association with lesion formation [26, 27]. To evaluate the significance of these two molecules, Cybulsky et al. crossed VCAM-1^D4D^ deficient mice (in which the fourth immunoglobulin domain, which is responsible for *α*_4_ integrin binding and is disrupted and both the mRNA and protein expression of VCAM-1 are decreased to 2–8% of wild-type levels, without resulting in lethality) with LDL-deficient mice, generating* VCAM *^D4D/D4D^ mice. These animals exhibited a marked decrease in lesion areas during early AS [25]. Similar results were obtained by Dansky using the ApoE^−/−^ mouse model [28]. In contrast, ICAM-1-deficiency did not affect lesion formation during early AS [25]. These studies suggested that VCAM-1 might play a dominant role in the initiation of AS.

In traditional Chinese medicine (TCM), Angong Niuhuang pill (ANP) is widely accepted as one of the most famous formulas used for the emergency clinical management of CVD [29]. The major constituents of ANP include* Bovis* Calculus Sativus, Moschus, Pulvis* Bubali* Comus Concentratus, Margarita,* Coptidis* Rhizoma, Realgar, Curcumae Radix, Borneolum Syntheticum,* Scutellariae* Radix, and* Gardeniae* Fructus, which synergistically exert pharmacological effects on central nervous system diseases such as stroke, coma, and centric fever [30]. Further, ANP can also have potential therapeutic effects on inflammatory diseases and can effectively reduce the number of inflammatory cells around hematomas and inhibit the expression of TNF-*α*, resulting in the inhibition of inflammation in rats with intracerebral hemorrhage [31]. Viral encephalitis comprises a typical intracranial inflammatory reaction and ANP can also decrease levels of TNF-*α* in the cerebrospinal fluid and exhibit a higher rate of efficacy than basic Western treatment. Therefore, given the tight association between inflammation and AS, ANP has the potential to provide novel breakthroughs for treating this condition [32]. As expected, Fu et al. demonstrated that ANP can significantly improve pathological indicators in a high-fat and vitamin D3-induced AS mouse model by inhibiting platelet accumulation and decreasing the low-density lipoprotein cholesterol/high-density lipoprotein cholesterol (LDL-C/HDL-C) ratio and total cholesterol content, among other parameters [29]. However, how ANP exerts its anti-inflammatory functions in AS is still unknown. To further investigate the protective mechanism of ANP during early AS, we performed a series of studies from an anti-inflammation perspective using the high-fat diet-induced ApoE^−/−^ mouse model of early AS.

## 2. Materials and Methods

### 2.1. ANP Preparation

ANP (Lot: WA0076) was prepared by Guangzhou Baiyunshan Zhongyi Pharmaceutical Co., Ltd. (Guangzhou, China) according to the standard operating procedures. Briefly, Cinnabaris, Margarita, and Realgar were ground or pulverized to very fine powders.* Coptis chinensis* Franch.,* Scutellaria baicalensis Georgi*,* Gardenia jasminoides* Ellis, and* Curcuma wenyujin *Y. H. Chen and C. Ling were pulverized to a fine powder.* Bovis* Calculus Sativus, powered buffalo horn extract,* Moschus*, and Borneolum Syntheticum were triturated with these powders, sifted, and mixed well. Refined honey was mixed to make 600 big honeyed pills (Editorial Committee of Pharmacopoeia of Ministry of Health PR China, 2015). All voucher specimens were deposited in the specimen room of Guangzhou Baiyunshan Zhongyi Pharmaceutical Co., Ltd., Guangzhou, China. The fingerprint chromatography of ANP was used for verification of the contents within the powders; for details please see the supporting information.

### 2.2. Reagents

Simvastatin (Lot: 20170101) was purchased from Shandong Xinqi Pharmaceutical Co., Ltd. (Shandong, China). Goat anti-rabbit secondary antibody (Lot: 040818180510), ICAM-1, and VCAM-1 primary antibodies (Lots: 7301248 and 7301143) were purchased from Affinity Biosciences Co., Ltd. (OH, USA). TRIzol reagent (Lot: 80802) was purchased from Thermo Fisher Science Co., Ltd. (MA, USA). PrimeScript RT reagent kit (Lot: AK4601) was from Takara Co., Ltd. (Tokyo, Japan). High-fat and high cholesterol feed (composition: 20% sucrose, 1.2% cholesterol, 0.2% sodium cholate, 10% casein, 0.6% calcium hydrogen phosphate, 0.4% fine aggregate, 0.4% premix, and 52.2% basic feed) was provided by the Medical Science Experimental Animal Center (Guangdong, China). PE anti-mouse CD25 (Lot: B193989), APC anti-mouse CD127 (Lot: A7R34), PE/Cy7 anti-mouse CD4 (Lot: B214650), Alexa Fluor® 488 anti-mouse IL-17A (Lot: B193887), APC Rat IgG2a, *κ* Isotype Ctrl (Lot: B207024), PE Rat IgG2b, *κ* Isotype Ctrl (Lot: B236195), Alexa Fluor® 488 Rat IgG1, *κ* Isotype Ctrl (Lot: B187687), PE/Cy7 Rat IgG2a, *κ* Isotype Ctrl (Lot: B236195), Cell Activation Cocktail (with Brefeldin A) (Lot: B233476), RBC Lysis Buffer (10×) (Lot: B29740), Fixation Buffer (Lot: S07063M), and Intracellular Staining Permeabilization Wash Buffer (10×) (Lot: B235807) were purchased from American BioLegend Co., Ltd. (CA, USA).

### 2.3. Animals and Model Establishment

Specific pathogen-free healthy male C57BL/6J mice (weight: 18 ± 2.0 g, n = 12) and SPF healthy male ApoE^−/−^ mice (weight: 18 ± 2.0 g, n = 25) were provided by the Peking University Health Science Center (Department of Laboratory Animal Science) in Beijing, China (Certificate no. SCXK (Beijing) 2011-0012).

SPF healthy male ApoE^−/−^and C57BL/6J mice were administered a normal diet for 10 days. The ApoE^−/−^ mice were then administered high-fat and high cholesterol feed and water ad libitum for 8 weeks, to represent the early stage of AS progression.

### 2.4. Drug Administration

At 4 weeks of age and after 10 days of adaptive feeding, 12 basic-diet-fed male C57BL/6J mice were randomly divided into a control group (0.1 mL/10 g normal saline) and an ANP-treated group (0.5 g/kg, 0.1 mL/10 g). Thirty-two male ApoE^−/−^ mice were randomly divided into five groups including a normal diet-fed negative control group (0.1 mL/10 g normal saline) and four high-fat-induced early AS model groups as follows: vehicle- (0.1 mL/10 g normal saline), low-dose ANP- (0.25 g/kg, 0.1 mL/10 g), high-dose ANP- (0.50 g/kg, 0.1 mL/10 g), and simvastatin- (0.35 mg/kg, 0.1 mL/10 g) treated groups. Each group comprised 6–7 mice with all mice intragastrically administered the designated drug, every other day for 8 weeks. All drugs were dissolved in distilled water to a specific concentration immediately before administration.

### 2.5. Sample Collection

Blood serum: after 8 weeks of drug administration, whole blood from the eyeball was collected from mice following anesthesia with 1% sodium pentobarbital by intraperitoneal injection. Whole blood was then transferred to a 1.5-mL vacuum tube and centrifuged for 8 min (4°C, 3000 rpm). The supernatant was collected and stored at −20°C.

Splenocyte suspension: following the collection of blood serum, mice were sacrificed by cervical vertebra dislocation and sterilized for 3 min in a beaker with 75% (v/v) ethanol. The spleen of the mice was dissected on a clean bench and placed in a petri dish with 7 mL of RPMI1640 medium. This tissue was then washed several times in medium using a syringe. The splenocyte suspension in the medium was then collected and the spleen was wrapped in silver foil and stored in liquid nitrogen.

Tissue: the whole-length aorta was rapidly removed for further sample collection. The entire aortic arch was separated, and a section of the aorta was placed in a 1.5-mL vacuum tube filled with 4% paraformaldehyde and stored at 4°C. The remaining sections of the aorta were wrapped in silver foil and stored in liquid nitrogen.

### 2.6. Pathological Observation

Tissues were fixed in a 4% paraformaldehyde solution and embedded in paraffin after dehydration. Pathological sections were prepared for oil red O staining and photographed using a microscope.

### 2.7. Analysis of Biochemical Indices

The serum levels of glucose, total cholesterol, triglyceride, LDL-C, and HDL-C were measured using an automatic biochemical analyzer in accordance with the manufacturer's instructions.

### 2.8. qPCR Analysis

The mRNA levels of* IL-6*,* TGF-β1*,* IL-17A*, and* IL-10* in the spleen and aorta, as well as* MCP-1*,* MCP-2*,* MCP-3*,* CCR2*, and* CXCR3* in the aorta, were analyzed by qPCR. RNA from the spleen and aorta of mice was extracted using TRIzol reagent. The concentration and purity of RNA was analyzed using a microdetector. The RNA was then reverse transcribed to cDNA according to the manufacturer's recommendations for the PrimeScript RT reagent kit. The cDNA sample was net used for qPCR analysis.

*Primer Information*

**Table:** 

IL-6:	Forward primer	GAGGATACCACTCCCAACAGACC
	Reverse primer	AAGTGCATCATCGTTGTTCATACA
TGF-*β*1:	Forward primer	TGACGTCACTGGAGTTGTACGG
	Reverse primer	GGTTCATGTCATGGATGGTGC
IL-17A:	Forward primer	TCTCTGATGCTGTTGCTGCT
	Reverse primer	CGTGGAACGGTTGAGGTAGT
IL-10:	Forward primer	GACAACATACTGCTAACCGACTC
	Reverse primer	TCACTCTTCACCTGCTCCACTG
MCP-1:	Forward primer	TTCACCAGCAAGATGATCCCA
	Reverse primer	TCCTTCTTGGGGTCAGCACA
MCP-2:	Forward primer	TCTACGCAGTGCTTCTTTGCC
	Reverse primer	AAGGGGGATCTTCAGCTTTAGTA
MCP-3:	Forward primer	TCTGTGCCTGCTGCTCATAG
	Reverse primer	CTTTGGAGTTGGGGTTTTCA
CCR2:	Forward primer	ACAGCTCAGGATTAACAGGGACTTG
	Reverse primer	ACCACTTGCATCCACACATGAC
CXCR3:	Forward primer	AGAATCATCCTGGTCTGAGACAA
	Reverse primer	AAGATAGGGCATGGCAGC

### 2.9. Immunohistochemistry (IHC) Analysis

Protein levels of ICAM-1 and VCAM-1 in the aortic arch were analyzed by IHC. The aorta of mice was fixed in a 4% paraformaldehyde solution and embedded in paraffin after dehydration. Frozen sections were also fixed for further antigen retrieval and for the inhibition of endogenous peroxidase. Sections were incubated with the primary antibody and the secondary antibody after blocking with a 5% solution of skim milk. The targeted antigens were detected by DAB staining. The nucleus was dyed and dehydrated, and the sections were imaged and photographed using a microscope. Statistical analysis was performed using Image Pro Plus (MD, USA), and results were considered statistically significant at a P value < 0.05.

### 2.10. Flow Cytometric Analysis

The ratio of Th17 to Treg cells in splenocyte suspensions was analyzed by flow cytometry. The suspension was stimulated with 10 *μ*L of Cell Activation Cocktail (with Brefeldin A) and mixed. After incubation at 37°C for 6 h, the sample was centrifuged, and the precipitate was suspended in 1×RBC. The suspension was then centrifuged and the precipitate resuspended in PBS twice. After antibody labeling of surface proteins, the sample was incubated in the dark for 20 min followed by centrifugation (2000 rpm). The cells in the precipitate were then fixed with fixation buffer, followed by incubation in the dark for 20 min. The sample was again centrifuged and suspended in 1×Permeabilization Wash Buffer. The cell was then washed twice and labeled with an immunofluorescent antibody. After incubation in the dark for 20 min, the sample was washed using 1×Permeabilization Wash Buffer twice and suspended in PBS prior to flow cytometric detection.

### 2.11. Statistical Analysis

Statistical significance was determined using SPSS13.0. Values are presented as means ± SEM. Enumerated data were expressed as sample percentage or size. The comparison between groups was determined by variance analysis of multiple sample means. The chi-square test was adopted to analyze the sample percentage or size. Results were considered statistically significant at a P value < 0.05.

## 3. Results

### 3.1. Establishing the AS Mouse Model

**Figure 1:**
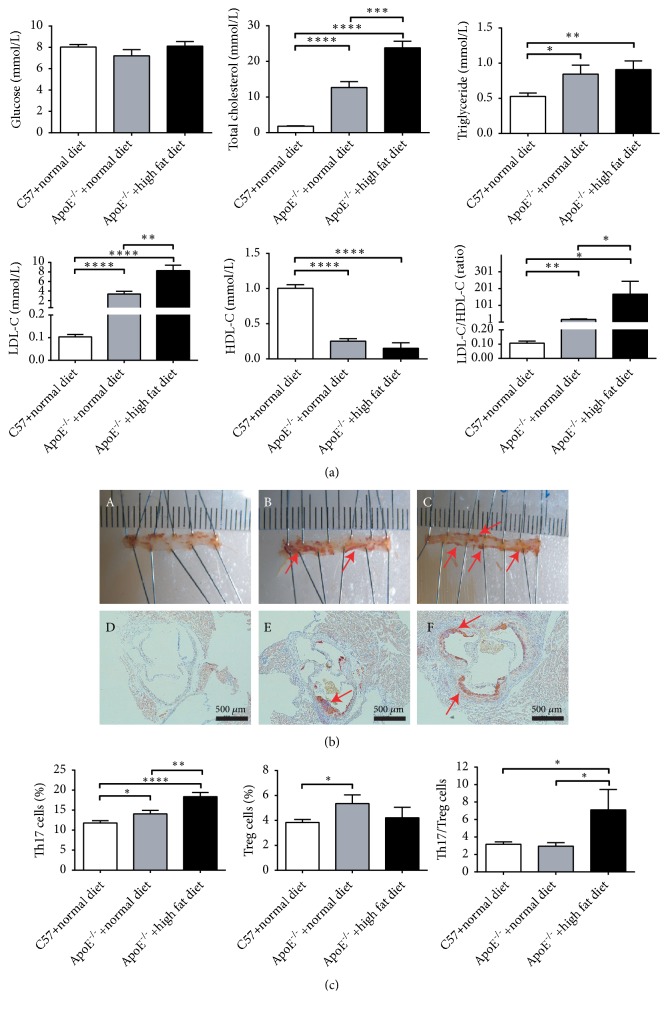
*A high-fat diet promotes the establishment of atherosclerosis (AS) in ApoE* ^*−/−*^ *mice.* (a). Serum levels of glucose, total cholesterol, triglyceride, LDL-C, and HDL-C in C57BL/6J mice fed a normal diet and in ApoE^−/−^ mice fed a normal or high-fat diet. Each group contained six mice. (b) Oil red O staining of aorta (A, B, and C) and aortic arch (D, E, and F). A and D: C57BL/6J mice with normal diet; B and E: ApoE^−/−^ mice fed a normal diet; C and F: ApoE^−/−^ mice administered a high-fat diet. Red arrows indicate the aortic arch and aortic staining sites. (c) The percentage of splenic Treg and Th17 cells, as detected by flow cytometry. The data are presented as means ± SEM. *∗*P < 0.05; *∗∗*P < 0.01; *∗∗∗*P < 0.001; *∗∗∗∗*P < 0.0001; results were considered statistically significant at P < 0.05.

The ApoE^−/−^ mouse model is considered the best model for experimental AS and was selected in our study. AS was induced by administering a high-fat diet to ApoE^−/−^ mice. To evaluate this model, the serum levels of glucose, total cholesterol, triglyceride, LDL-C, and HDL-C in C57BL/6J mice fed a normal diet and ApoE^−/−^ mice treated with either a normal or high-fat diet were determined. Compared to the concentrations of total cholesterol, triglyceride, and LDL-C in C57BL/6J mice, these parameters were significantly increased in ApoE^−/−^ mice fed a high-fat or normal diet. More importantly, the high-fat diet accelerated the increase in levels of the aforementioned markers. LDL-C was markedly increased in ApoE^−/−^ mice and the LDL-C/HDL-C ratio was as high as 150 in these animals when they were fed a high-fat diet. No significant difference was observed in glucose levels between ApoE^−/−^ and C57BL/6J mice (Figure 1(a)). In addition, the accumulation of lipids throughout the aorta and aortic arch of each experimental group was examined by oil red O staining. The plaque areas in ApoE^−/−^ mice were more extensive than those in C57BL/6J mice; further, lipid deposition in ApoE^−/−^ mice fed a high-fat diet was higher than that in ApoE^−/−^ mice fed a normal diet (Figure 1(b)). Th17/Treg imbalances are an important indication of AS. To assess the polarization of splenic lymphocytes, we analyzed splenic single-cell suspensions by flow cytometry. As shown in Figure 1(c), the frequency of Th17 cells was significantly increased in ApoE^−/−^ mice; no significant difference in Treg cells was observed for ApoE^−/−^ mice fed high-fat diet. Taken together, the ratio of Th17/Treg cells was elevated in ApoE^−/−^ mice fed a high-fat diet. These results therefore demonstrated that we had successfully established an AS mouse model.

### 3.2. ANP Toxicity Assessment

**Figure 2:**
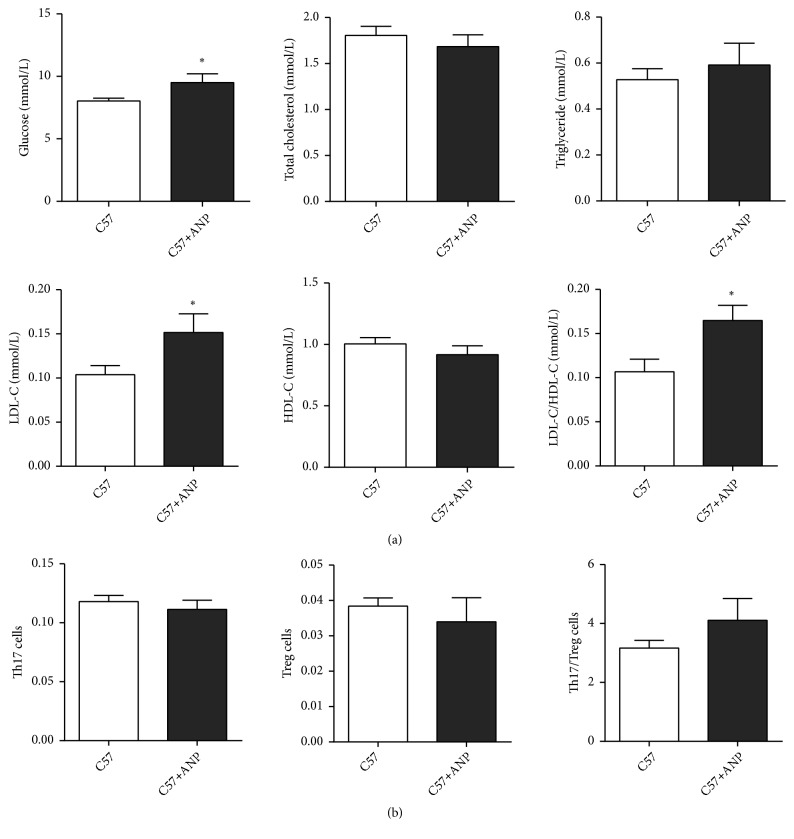
*Effect of Angong Niuhuang pill (ANP) on biochemical indices and the polarization of splenic lymphocytes in C57BL/6J mice.* (a) Serum levels of glucose, total cholesterol, triglyceride, LDL-C, and HDL-C in C57BL/6J mice with or without ANP treatment. (b) Percentage of splenic Treg and Th17 cells, as detected by flow cytometry. Each group contained six mice and the dose of ANP administered was 0.5 g/kg. The data are presented as means ± SEM, and results were considered statistically significant at *∗*P < 0.05.

The toxicity of ANP was then assessed in the C57BL/6J mice, and the serum levels of glucose, total cholesterol, triglyceride, LDL-C, and HDL-C, as well as the polarization of splenic lymphocytes, were detected. As shown in Figure 2(a), the levels of total cholesterol, triglyceride, and HDL-C were not different in C57BL/6J mice, with or without ANP treatment. The levels of glucose and LDL-C and the ratio of LDL-C/HDL-C increased slightly in C57BL/6J mice treated with ANP; however, the slight increase observed was significant compared to those in C57BL/6J mice without ANP treatment. Splenic lymphocyte polarization analysis indicated that there was no significant difference between Th17 and Treg cells (Figure 2(b)). These results suggested that ANP only slightly increased the levels of glucose and LDL-C, indicating its low toxicity.

### 3.3. Effect of ANP on Biochemical Indices

**Figure 3:**
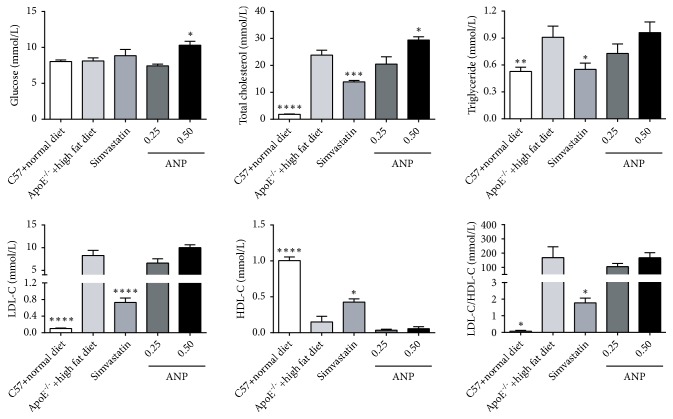
*Effect of Angong Niuhuang pill (ANP) on serum levels of glucose, total cholesterol, triglyceride, LDL-C, and HDL-C.* The groups of ANP (0.25 and 0.5 g/kg mouse body weight) treatment contained seven mice, whereas the other groups contained six mice. Data from ApoE^−/−^ mice administered a high-fat diet was set as the reference. The data are presented as the means ± SEM, and *∗*P < 0.05; *∗∗*P < 0.01; *∗∗∗*P < 0.001; *∗∗∗∗*P < 0.0001; results were considered statistically significant at P < 0.05.

To evaluate the effect of ANP on the alleviation of AS, ApoE^−/−^ mice were treated with a low dose (0.25 g/kg) or a high dose (0.50 g/kg) of ANP or simvastatin (3.5 mg/kg). Serum levels of glucose, total cholesterol, triglyceride, LDL-C, and HDL-C were then detected. Compared to levels in ApoE^−/−^ mice fed a high-fat diet, ANP (0.50 g/kg) upregulated glucose and total cholesterol levels. No significant difference in the levels of triglyceride, LDL-C, and HDL-C was observed in ApoE^−/−^ mice, whether they were treated with a low or high concentration of ANP. However, simvastatin reduced the levels of total cholesterol, triglyceride, and LDL-C and the ratio of LDL-C/HDL-C and increased levels of HDL-C (Figure 3).

### 3.4. Effect of ANP on the Th17/Treg Ratio

**Figure 4:**
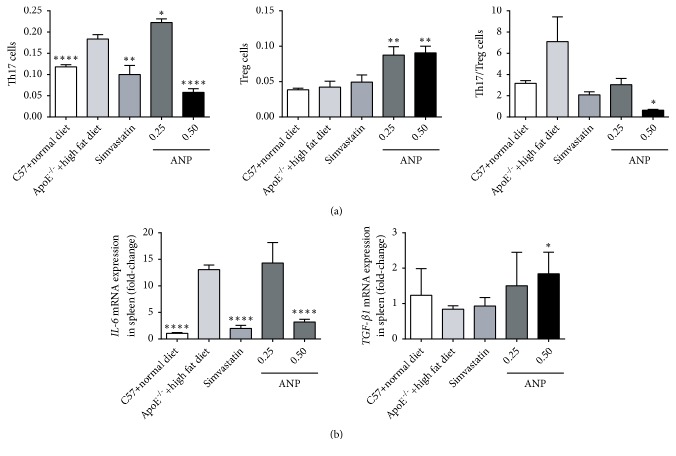
*Effect of Angong Niuhuang pill (ANP) on the polarization of splenic lymphocytes and IL-6 and TGF-β1 cytokines.* (a) Effect of ANP on the percentage of splenic Treg and Th17 cells, as detected by flow cytometry. (b) mRNA levels of* IL-6* and* TGF-β1* were determined by qPCR. The data for ApoE^−/−^ mice fed a high-fat diet was set as the reference. The data are presented as means ± SEM. *∗*P < 0.05; *∗∗*P < 0.01; *∗∗∗∗*P < 0.0001; results were considered statistically significant at P < 0.05.

ANP had a strong effect on the polarization of splenic lymphocytes. Administering a high dose of ANP downregulated Th17 cells and upregulated Treg cells. Further, the ratio of Th17/Treg cells was 4-fold and 3-fold lower in C57BL/6J mice fed a normal diet and in ApoE^−/−^ mice treated with simvastatin, respectively (Figure 4(a)). Cytokines, and especially IL-6 and TGF-*β*1, are the most important determinants of the fate of CD4 T cells. Therefore, we performed qPCR assays to detect the mRNA levels of* IL-6* and* TGF-β1* in the spleen. As shown in Figure 4(b), in the spleens of ApoE^−/−^ mice fed a high-fat diet, an increase of more than 10-fold was observed in the mRNA expression of* IL-6*, whereas the mRNA level of TGF-*β*1 decreased with no significant difference. After treatment with ANP (0.50 g/kg), the mRNA expression level of* IL-6* or* TGF-β1* was downregulated or upregulated, respectively, to almost control levels. Nevertheless, low-dose ANP did not have any effect. Simvastatin achieved a similar effect on the regulation of* IL-6* mRNA; however, the modulation of* TGF-β1* mRNA expression was not observed.

### 3.5. Effect of ANP on the mRNA Expression of Cytokines in the Aorta

**Figure 5:**
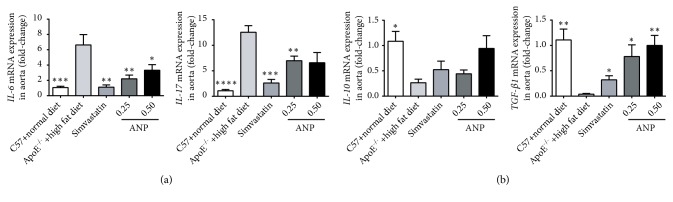
*Effect of Angong Niuhuang pill (ANP) on mRNA expression of IL-6, IL-17, IL-10, and TGF-β1 in the aorta.* (a) mRNA levels of the proinflammatory cytokines IL-6 and IL-17. (b) mRNA levels of anti-inflammatory cytokines* IL-10* and* TGF-β1*. The mRNA levels in ApoE^−/−^ mice fed a high-fat diet were used as references. *∗*P < 0.05; *∗∗*P < 0.01; *∗∗∗*P < 0.001; P *∗∗∗∗* < 0.0001; P < 0.05 indicates significant difference.

Cytokines play important roles in all stages of AS and have a profound influence on the pathogenesis of this disease. The proinflammatory cytokines IL-6 and IL-17 and the anti-inflammatory cytokines TGF-*β*1 and IL-10 were thus determined in the aorta. In the high-fat-diet-induced ApoE^−/−^ mice, both IL-6 and IL-17 levels were increased, and low and high doses of ANP partly restored IL-6 expression. Low-dose ANP also significantly downregulated IL-17 expression, whereas high-dose ANP did not produce a similar result (Figure 5(a)). In contrast, levels of the anti-inflammatory cytokines TGF-*β*1 and IL-10 decreased during the development of AS; however, a reversal to control levels was attained using ANP. Nevertheless, for IL-10, this effect was not statistically significant (Figure 5(b)).

### 3.6. Effect of ANP on mRNA Expression of Chemokines and Chemokine Receptors in the Aorta

**Figure 6:**
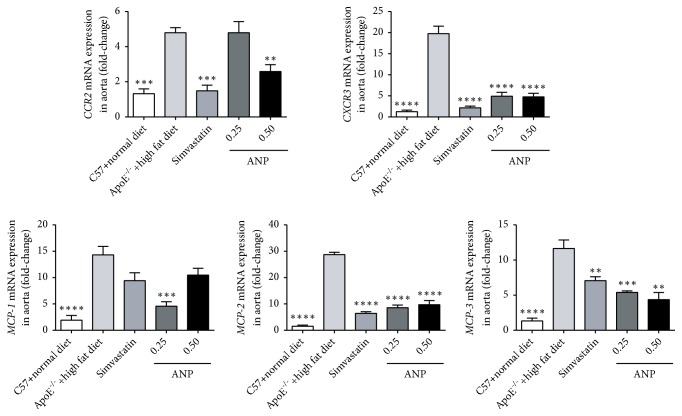
*Effect of Angong Niuhuang pill (ANP) on the mRNA expression of CCR2, CXCR3, and MCPs in the aorta.* The mRNA levels were detected using qPCR, and mRNA levels in ApoE^−/−^ mice fed a high-fat diet were set as the reference. *∗∗*P < 0.01; *∗∗∗*P < 0.001;*∗∗∗∗*P < 0.0001; results were considered statistically significant at P < 0.05.

Given the importance of chemokines for the development of AS, these molecules and their receptors including MCP1-3, CCR2, and CXCR3 were detected in the aorta by qPCR. As shown in Figure 6, compared to that in the C57BL/6J mouse group, the mRNA expression levels of all chemokines and their receptors in ApoE^−/−^ mice fed a high-fat diet were significantly increased (*P* < 0.001). Similar to that observed with simvastatin, treatment with high- or low-dose f ANP efficiently downregulated the mRNA levels of these markers, and these differences were significant, except for that for MCP-1. ANP (0.50 g/kg) decreased* MCP-1* mRNA levels; however, this was not significant. ANP (0.25 g/kg) can therefore significantly reduce* MCP-1* mRNA expression, almost to control levels observed in the C57BL/6J mice group. Overall, ANP effectively decreased these proinflammatory chemokines and their receptors.

### 3.7. Effect of ANP on CAM Expression

**Figure 7:**
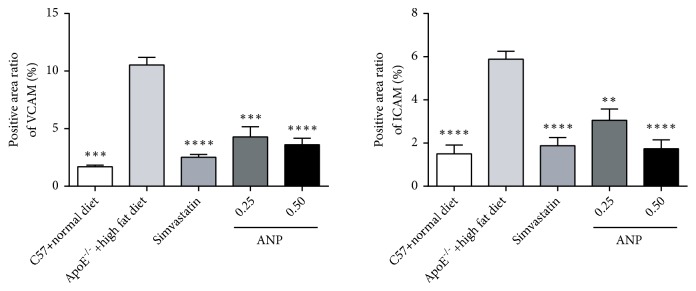
*Effect of Angong Niuhuang pill (ANP) on VCAM-1 and ICAM-1 expression in the aortic arch.* IHC was performed, and the positive area was analyzed to indicate the relative protein levels in the different groups. The values for ApoE^−/−^ mice fed a high-fat diet were used as references. *∗∗*P < 0.01; *∗∗∗*P < 0.001; *∗∗∗∗*P < 0.0001; P < 0.05 indicates a significant difference.

The effect of ANP on the expression levels of VCAM-1 and ICAM-1 in the aortic arch was then observed using IHC. Both VCAM-1 and ICAM-1 increased during the progression of AS; however, low- and high-dose ANP significantly decreased VCAM-1 and ICAM-1 expression. Similar changes were observed in positive control mice treated with simvastatin, and the effect of ANP on the regulation of VCAM-1 and ICAM-1 was able to counter that of simvastatin (Figure 7).

**Figure 8:**
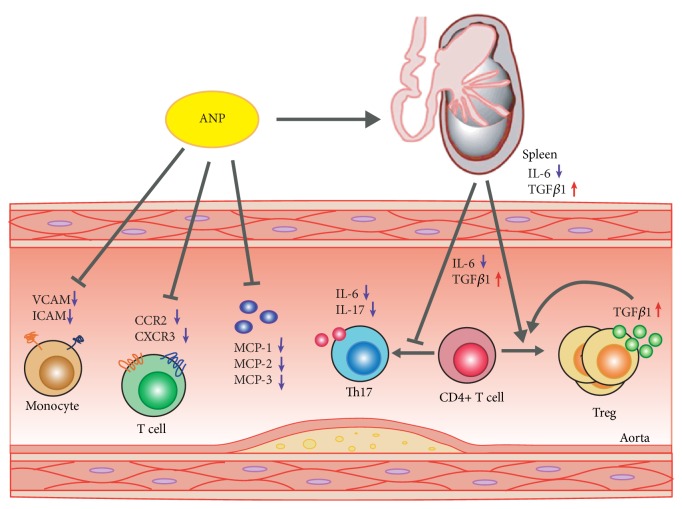
*Proposed model for the inhibition of early atherosclerosis (AS) progression by Angong Niuhuang pill (ANP) through the targeting of multiple aspects of inflammation.* ANP up- and downregulates splenic IL-6 and TGF-*β*1, respectively, jointly promoting CD4+ T cells to differentiate into Treg cells. Meanwhile ANP decreases IL-6, IL-17, CCR2, CXCR3, MCPs, and CAMs and increases TGF*β*1 in the aorta, thus, inhibiting early AS progression.

In conclusion, ANP prevents early AS progression by reducing both T lymphocyte inflammation and arterial wall inflammation, without promoting hyperlipidemia, which was found to be potentially equivalent to the effects of simvastatin. These results demonstrate that, by targeting inflammation, ANP is a more efficient drug formulation for treating or preventing early atherogenesis. Meanwhile, ANP functions through multiple targets (e.g., the Th17/Treg ratio, cytokines, chemokines, and CAMs), which play independent and additive roles in AS (Figure 8). Therefore, ANP is much more powerful than single target inhibitors or drugs.

## 4. Discussion

CVD, a widespread chronic disease, is a leading cause of death globally. Along with the acceleration of living rhythm and improved living standards, more factors can lead to an increased risk of CVD. These factors include but are not limited to lifestyle, high pressure, and dietary patterns. The age of individuals affected by CVD morbidity tends to be younger, which makes it even more concerning and suggesting that it is necessary to reduce the likelihood of its occurrence. There is an old Chinese saying, “Prevention is better than cure”; thus, the development of new, safe, and effective therapeutics or drug repurposing to prevent CVD progression during early onset is essential [33]. AS is known to culminate in CVD and inflammation plays a predominant role in atherogenesis [34]. Thus, regulating the related inflammatory pathways, especially during the early stage of AS, is an efficient way to prevent disease progression [35].

ANP, a popular TCM, is extensively used in clinical practice to treat CVD and has been approved by the CFDA (State Food and Drug Administration of China). However, little is known about its effects on inflammation in the early stage of AS. To better understand its effect during early atherogenesis, an early stage model of AS was established in this study. ApoE^−/−^ is one of the best studied AS models and can spontaneously develop this condition at a relatively slow rate [36]. To accelerate this process, in our study, ApoE^−/−^ mice were fed a high-fat diet. All biochemical indices and related parameters (serum contents of glucose, total cholesterol, triglyceride, HDL-C, LDL-C, and LDL-C/HDL-C) (Figure 1(a)), oil red O staining in the aortic arch (Figure 1(b)) and spleen and an analysis of the Th17/Treg ratio (Figure 1(c)) indicated that the early AS model was successfully established at 8 weeks.

ANP, a mixture of 11 herbs, contains a variety of phytochemicals, proteins, and polysaccharides; thus, confirming its toxicity is extremely important. High-dose ANP (0.5 g/kg)-treated C57BL/6J mice did not show any significant differences in biochemical parameters compared to those in the untreated group, proving that ANP is relatively safe for use with no apparent side effects.

The functional mechanism underlying the effects of ANP was further studied. Interestingly, the ANP and simvastatin (positive control) groups showed different patterns in biochemical indices. High-dose ANP had no effect on the serum content of triglyceride, HDL-C, LDL-C, and LDL-C/HDL-C, but upregulated glucose and total cholesterol levels, whereas simvastatin significantly increased the levels of HDL-C and decreased total cholesterol, triglyceride, LDL-C, and LDL-C/HDL-C levels. These results indicate that ANP might have a different mechanism from simvastatin during the early stage of AS. Previous studies demonstrated that several molecular and cellular inflammation pathways participate in atherogenesis [37]. Along with AS development, the Th17/Treg ratio increases gradually, and treatment with ANP can effectively restore this balance. Moreover, the expression levels of corresponding cytokines (IL-6 and TGF-*β*1) also showed the same pattern in the spleen and aorta. This suggests that cytokines can generate feedback control in different T lymphocyte subsets to maintain their homeostasis. It is worth mentioning that high-dose ANP (0.5 g/kg) was more potent in restoring the Th17/Treg ratio and regulating cytokine expression than simvastatin. This implies that ANP can potentially be used for intervention therapy during early AS. Moreover, ANP also alleviated arterial wall inflammation based on three relatively independent processes as follows: (1) inhibiting the mRNA expression of proinflammatory cytokines (*IL-6* and* IL-17*), increasing the expression of anti-inflammatory cytokines (*IL-10* and* TGF-β1*), and exerting a stronger effect (or at least comparable) than simvastatin, even at low dose; (2) downregulating the expression of chemokines and their receptors (MCPs/CCR2, CXCR3); (3) downregulating adhesion molecules (ICAM and VCAM). Combined with the results obtained from the oil red O staining analysis (data not shown), ANP did not have an obvious effect on the lesion area; however, it might inhibit the continuous growth of the area and negatively regulate the overall development of AS.

To fully understand the multitargeted anti-inflammatory effects of ANP during early AS, the following should be considered: (1) the roles that the Th17/Treg functional balance, cytokines, and chemokines play in the pathogenesis of ANP during early AS; (2) the effect of ANP on transcription factors of Th17 and Treg cells (e.g., ROR*γ*t and Foxp3) and their feedback regulation during the maintenance of the Th17/Treg balance; (3) the effect of ANP on other inflammatory cells (such as macrophages, dendritic cells, and T lymphocytes) in the early stage of plaque formation. Due to these limitations of this study, these questions will be the subject of further research.

In conclusion, ANP prevents early AS progression by reducing both T lymphocyte inflammation and arterial wall inflammation, without hyperlipidemia, and it is potentially equivalent to simvastatin based on these effects. These results demonstrate that by targeting inflammation, ANP is an efficient drug formulation for treating or preventing early atherogenesis. Meanwhile, ANP functions through multiple targets (e.g., the Th17/Treg ratio, cytokines, chemokines, and CAMs), which play independent and additive roles in AS. Therefore, ANP is much more powerful than single target inhibitors or drugs.

## Data Availability

The data used to support the findings of this study are available from the corresponding author upon request.

## Abbreviations

ANP: Angong Niuhuang pill
AS: Atherosclerosis
CAM: Cell adhesion molecule
CVD: Cardiovascular disease
HDL-C: High-density lipoprotein cholesterol
ICAMs: Intracellular adhesion molecules
IHC: Immunohistochemistry
IL: Interleukin
LDL-C: Low-density lipoprotein cholesterol
MCP: Monocyte chemoattractant protein
TCM: Traditional Chinese medicine
Th1: T helper cell 1
Treg: T regulatory
VCAMs: Vascular adhesion molecules.
